# Factors influencing the spatial extent of mobile source air pollution impacts: a meta-analysis

**DOI:** 10.1186/1471-2458-7-89

**Published:** 2007-05-22

**Authors:** Ying Zhou, Jonathan I Levy

**Affiliations:** 1 Department of Environmental Health, Harvard School of Public Health, Landmark Center 4^th ^Floor West, P.O. Box 15677, Boston, MA, 02215, USA

## Abstract

**Background:**

There has been growing interest among exposure assessors, epidemiologists, and policymakers in the concept of "hot spots", or more broadly, the "spatial extent" of impacts from traffic-related air pollutants. This review attempts to quantitatively synthesize findings about the spatial extent under various circumstances.

**Methods:**

We include both the peer-reviewed literature and government reports, and focus on four significant air pollutants: carbon monoxide, benzene, nitrogen oxides, and particulate matter (including both ultrafine particle counts and fine particle mass). From the identified studies, we extracted information about significant factors that would be hypothesized to influence the spatial extent within the study, such as the study type (e.g., monitoring, air dispersion modeling, GIS-based epidemiological studies), focus on concentrations or health risks, pollutant under study, background concentration, emission rate, and meteorological factors, as well as the study's implicit or explicit definition of spatial extent. We supplement this meta-analysis with results from some illustrative atmospheric dispersion modeling.

**Results:**

We found that pollutant characteristics and background concentrations best explained variability in previously published spatial extent estimates, with a modifying influence of local meteorology, once some extreme values based on health risk estimates were removed from the analysis. As hypothesized, inert pollutants with high background concentrations had the largest spatial extent (often demonstrating no significant gradient), and pollutants formed in near-source chemical reactions (e.g., nitrogen dioxide) had a larger spatial extent than pollutants depleted in near-source chemical reactions or removed through coagulation processes (e.g., nitrogen oxide and ultrafine particles). Our illustrative dispersion model illustrated the complex interplay of spatial extent definitions, emission rates, background concentrations, and meteorological conditions on spatial extent estimates even for non-reactive pollutants. Our findings indicate that, provided that a health risk threshold is not imposed, the spatial extent of impact for mobile sources reviewed in this study is on the order of 100–400 m for elemental carbon or particulate matter mass concentration (excluding background concentration), 200–500 m for nitrogen dioxide and 100–300 m for ultrafine particle counts.

**Conclusion:**

First, to allow for meaningful comparisons across studies, it is important to state the definition of spatial extent explicitly, including the comparison method, threshold values, and whether background concentration is included. Second, the observation that the spatial extent is generally within a few hundred meters for highway or city roads demonstrates the need for high resolution modeling near the source. Finally, our findings emphasize that policymakers should be able to develop reasonable estimates of the "zone of influence" of mobile sources, provided that they can clarify the pollutant of concern, the general site characteristics, and the underlying definition of spatial extent that they wish to utilize.

## Background

Given growing evidence of the health effects of traffic-related air pollution as well as of proximity to major roads [1-3], there has been growing interest in the concept of "hot spots", or more broadly, spatial gradients in exposures to and health risks from traffic-related air pollutants. Hot spots have been defined as locations where emissions from specific sources may expose individuals and population groups to elevated risks of adverse health effects – including but not limited to cancer – and contribute to the cumulative health risks of emissions from other sources in the area [4]. We more broadly consider the concept of the distance from a source at which such impacts would be observed, which we refer to as the "spatial extent" of the impacts.

Studies in the literature have used a variety of methods considering different pollutants in numerous settings to draw inferences about the spatial extent of mobile source air pollution. For example, GIS-based epidemiological investigations establish buffers around roadways and measure the cumulative traffic volume within a defined radius to link with a variety of health outcomes [5-8]. The radius most strongly associated with the health outcomes in question would be presumed to be the spatial extent of the impacts of the source, although the GIS measures are only occasionally able to be compared with measured pollutant concentrations (and then with only a subset of traffic-related pollutants) [9,10], and the radius of impact would theoretically differ by pollutant.

Other studies have monitored concentrations of a number of pollutants at varying distances from roadways [11,12] and compared these concentrations with background levels of the pollutant, often with the implicit assumption that the spatial extent of the source extends until the point at which the concentrations are not significantly different from background. Yet another approach for understanding the spatial extent involves modeling the health risks associated with the source in question. In the case of cancer risk assessment, studies have estimated the incremental health risks at different distances from the mobile source of interest [13,14], which were then compared with a predetermined risk threshold (i.e., a one in a million lifetime risk) to determine the spatial extent of influence. This approach will clearly have the same meteorological and site influences as the monitoring or GIS-based approaches, but the spatial extent in this case will also be dependent on assumptions in the risk assessment (i.e., the dose-response function) as well as on the threshold value selected.

Clearly, conclusions about the spatial extent of impact will be influenced by both the methodology used and factors more generally related to pollutant fate and transport, such as source strength, pollutant characteristics (i.e., reactivity, particle diameter), meteorological factors (i.e., wind speed and direction), or site characteristics (i.e., presence/absence of a street canyon). The combination of these factors has led to substantial variability in conclusions across studies from within tens of meters [15] to tens of kilometers or more [13].

At present, it is unclear which of the above factors contributes most significantly to the variability in spatial extent estimates, leading to some confusion and limiting generalizable insights from a public policy or planning perspective. For example, a stakeholder may wish to have preliminary insight about whether a roadway expansion would have deleterious effects on nearby residential populations or whether more intervention is needed on an existing port. The variability in the literature makes it difficult to determine whether a defined radius of concern is adequately protective, too restrictive, or not protective enough.

In this study, we conduct a quantitative meta-analysis of the published literature on the spatial extent of mobile source air pollution, with the objective of determining which factors best explain variability in spatial extent estimates. We supplement and provide further context for the interpretation of this meta-analysis by estimating spatial extent using a variety of definitions and parametric values within a simplified dispersion model. We use this information to draw conclusions about the current knowledge base and the spatial extent under various circumstances, with the overarching aim to determine whether it is plausible to construct representative spatial extent values for specific traffic-related air pollutants in unstudied settings.

## Methods

### Search criteria

In January 2006, we searched the Science Citation Index database for publications between 1997 and 2005 with keywords related to the spatial extent of mobile source related air pollution. We considered as relevant studies about on-road vehicles, construction engines/equipment, ports, and locomotives. We focused our search on four significant air pollutants related to mobile sources: particulate matter (PM), carbon monoxide (CO), benzene, and nitrogen oxides (NO_x_). CO and benzene represent non-reactive gaseous pollutants, while NO and NO_2 _represent gaseous pollutants that undergo rapid chemical reactions after being emitted. We include PM and particularly fine particles (PM_2.5_) because they pose a health risk [16,17] and because spatial patterns may differ in important ways for particles of different size fractions (i.e., ultrafine particles vs. particle mass).

Keywords used include

• Air pollution related terms such as "air pollution", "automobile exhaust", "particulate matter", "elemental carbon", "carbon monoxide", "benzene", "nitrogen oxides", and "hydrocarbons" as well as any variations of these terms (e.g., PM instead of particulate matter, NOx or oxides of nitrogen instead of nitrogen oxides);

• Spatial extent-related terms: e.g., "distance", "spatial extent", "spatial variability" or "spatial variation";

• Mobile source-related terms such as "highway", "traffic", "mobile source", "ferry", "marine vessel", "locomotive" as well as any variations of these terms

Among articles that met the search criteria, we excluded those studies that did not focus on air pollution dispersion from mobile sources (e.g., studies on mobile source emission factors, point sources). We also excluded studies with distance or spatial variation used in a general sense or as a continuous regression variable without conclusions about the spatial extent. We included a few earlier publications (before 1997), if they themselves satisfy the above criteria (except for the year of publication) and were cited by the publications which satisfied the above criteria.

In addition to the peer-reviewed literature, we collected government reports that focused on the spatial extent of mobile source (e.g., automobile, ferry, locomotive) air pollution. These studies provide either data on the spatial extent or an indication of how the literature has been interpreted in a policy context. Since there is no search database for reports that span a variety of government agencies (including federal and state agencies), we collected information through a targeted internet search on specific agency websites and from a previous report by Environmental Defense [18]. The reports reviewed here were not meant to be inclusive of all governmental reports on this topic, but rather provide a representative sample.

### Data gathering

From the identified studies, we extracted information about significant factors that would be hypothesized to influence the spatial extent within the study. This included the pollutant, background concentration, emission rate, and meteorological factors, as well as the site and season of the study, the methodology of the study, and its implicit or explicit definition of spatial extent. It is worth noting that most of the studies reviewed do not directly focus on spatial extent. Instead, information on spatial extent is provided as an ancillary part of the study findings. Therefore, not all information is available or meaningful for all studies. We either contacted the authors of these studies for clarification/additional information or used our judgments to determine proxies of these factors in cases where direct information was not available. Below, we briefly describe the rationale for each factor and our data extraction methodology.

#### Study type

Five types of studies potentially satisfy the search criteria: monitoring, air dispersion modeling, land use regression, epidemiology, and biomonitoring studies. In addition, some studies combine more than one of these study types (hybrid studies). Monitoring and air dispersion modeling studies directly provide information on the pollutant concentration gradient and the resulting spatial extent. Biomonitoring studies, which use organisms to assess or monitor environmental conditions (often analysis of trace elements in plants or plant species composition near the source of interest), are also directly informative, albeit with an indirect measurement methodology.

Land use regression model studies predict pollutant concentrations at a given site based on surrounding traffic and land use characteristics within various radii (or buffers) around the site [19]. This type of study is receptor-based rather than source-based, so is not directly informative about the spatial extent of mobile source impact, but the variables chosen for the final regression model can provide some insight on this issue. Similarly, epidemiological studies provide information on the distance from a roadway most strongly associated with health outcomes, an indirect measure of spatial extent. However, epidemiological studies which find no association between increased health risk and mobile source proximity provide little insight about the spatial extent of air pollution, as a null association could be related to many other factors (i.e., a lack of a causal association between the pollutant and health outcome in question).

For the quantitative meta-analysis, we created a categorical variable that indicated whether a study used monitoring/biomonitoring, dispersion modeling, epidemiology, or land-use regression.

#### Definition of spatial extent

The various spatial extent definitions used by different studies are generally based on absolute or relative comparisons. For relative comparisons, downwind concentrations as a percentage of a defined reference point concentration are compared with a cutoff value to determine spatial extent. The most commonly used reference point is the maximum concentration measured at the location nearest to the mobile source under study. The cutoff values used in the studies reviewed range from 50 percent of maximum concentration [15] to 10 percent [20,21]. For absolute comparisons, downwind pollutant concentrations or health risks are compared directly with threshold values or measurements upwind or at other distances to determine whether there are significant differences. The threshold values include health risk values such as 1 in a million [13,22] or incremental concentrations such as 0.01 ppm for NO_2 _[23]. Other approaches include selection of significant covariates in land-use regression models or epidemiological studies.

For the meta-analysis, we created a categorical variable indicating whether the study estimated the percentage decrease from a maximum concentration, a significant absolute difference from background levels, or all other approaches. These are broad categories with significant variability within each category, but we lacked the statistical power to address the nuances in these spatial extent definitions within our meta-analysis. This issue is addressed within our dispersion modeling case study.

#### Pollutant characteristics

Pollutant type will clearly influence the spatial extent, based both on chemical properties and background concentrations. For relatively inert pollutants such as CO, downwind concentrations mainly decrease through dilution with ambient air. For more reactive pollutants, their concentration profiles can also be influenced by the rate of chemical reactions. For example, NO reacts with ambient ozone to form NO_2 _near the emission source. For NO, the combination of the reaction and the dilution in the surrounding air mass results in a rapid decrease in concentration with downwind distance. For NO_2_, on the other hand, the dominant formation process slows down its dilution and the concentrations decrease at a gradual rate. Both the intrinsic reaction rate and the abundance of substances involved in the reactions such as O_3 _can play an important part in the concentration distribution. These factors would imply that there could be important seasonal influences on the spatial extent of reactive pollutants, so we gathered information about season of study where available.

Particulate matter is involved in different processes depending on particle size. For particles larger than about 1 μm, turbulent diffusion and gravitational settling are the dominant processes, whereas, for particles smaller than 0.1 μm, Brownian diffusion becomes increasingly important [24]. Due to coagulation processes wherein aerosol particles collide with one another and adhere to form larger particles [25], there will be a continuous decrease in number concentration coupled with an increase in particle size. For smaller particles (e.g., particles smaller than 0.1 μm), the combination of coagulation and dilution results in a rapid decrease in concentration with downwind distance. For larger particles, the formation through coagulation slows down the dilution and the concentrations decrease at a gradual rate. Thus, particle size and consideration of number vs. mass are key factors to consider.

Related to pollutant type is the magnitude of background concentrations relative to concentrations attributable to the source in question. For monitoring studies, the concentrations measured at different distances from the roadway include both background concentrations and the incremental contribution from the mobile source of interest. Therefore, the resulting concentration profile would be very different for two pollutants with and without significant background concentrations, even if the same amount of them are emitted from the source under study and they have similar dispersion characteristics. For example, suppose that the emissions from the source of interest result in a concentration increase of 1 μg/m^3 ^at 10 m and 0.1 μg/m^3 ^at 100 m for both pollutant A and B. If A has no background concentration while B has a uniform background concentration of 10 μg/m^3^, then it appears that the measured concentration of A at 100 m is only 10% of that at 10 m (90% decrease) while for B, the concentration at 100 m is still 92% of that at 10 m (less than 10% decrease), even though the source contribution is the same. This will have an influence on statistical significance tests and any "spatial extent" definitions based on concentration ratios.

Among the pollutants we focus on, CO, benzene and NO_x _have relatively low background concentrations. Mobile sources (both on-road and nonroad) are responsible for about 80 percent of benzene and CO, and more than half of all NO_x _emissions in the United States [26]. PM_2.5 _is primarily related to regional transport with the majority contributed by non-mobile sources [26], but ultrafine particles (smaller than 0.1 μm) and elemental carbon/black smoke are more closely related to local sources. For the meta-analysis, we develop a categorical variable combining pollutant type and background concentrations. We categorize each study as either "inert pollutant, high background" (PM mass without background removed in the analysis), "inert pollutant, low background or background removed" (CO, benzene, EC/black smoke, PM mass with background removed in the analysis), "reactive pollutant, near-source removal" (NO, ultrafine particles), "reactive pollutant, near-source formation" (NO_2_). Of note, background concentrations are generally not addressed in air dispersion modeling studies, but are explicitly reported in monitoring studies in which the comparison between the downwind concentrations and that of the background is used to define spatial extent.

#### Emission rate

If we assume a linear relationship between concentration and emission rate, an increased emission rate will increase the spatial extent of the impacts, if the spatial extent is defined as the distance at which a risk threshold is reached, the distance before which the concentration change is greater than a predefined value, or statistical significance tests are being used. Few studies directly report emission rates, but there are multiple useful proxies. Most monitoring studies use higher traffic counts to indicate higher emission rates, so we created a categorical variable for traffic count above/below the median across studies reporting traffic counts.

#### Meteorological factors

The importance of meteorological factors is apparent when considering a simple Gaussian equation [27] for estimating the concentrations downwind of a continuously emitting infinite line source for relatively inert pollutants: C=2Q2πUσz
 MathType@MTEF@5@5@+=feaafiart1ev1aaatCvAUfKttLearuWrP9MDH5MBPbIqV92AaeXatLxBI9gBaebbnrfifHhDYfgasaacH8akY=wiFfYdH8Gipec8Eeeu0xXdbba9frFj0=OqFfea0dXdd9vqai=hGuQ8kuc9pgc9s8qqaq=dirpe0xb9q8qiLsFr0=vr0=vr0dc8meaabaqaciaacaGaaeqabaqabeGadaaakeaacqWGdbWqcqGH9aqpdaWcaaqaaiabikdaYiabdgfarbqaamaakaaabaGaeGOmaidcciGae8hWdahaleqaaOGaemyvauLae83Wdm3aaSbaaSqaaiabdQha6bqabaaaaaaa@3863@, in which C is the downwind concentration (μg/m^3^), Q is the source strength per unit distance (μg/(m•s)), U is the average wind speed (m/s), and σ_z _is the vertical dispersion coefficient (m).

Wind speed determines the extent to which pollutants are initially diluted, with the inverse relationship between the wind speed and concentration given in the Gaussian equation. Wind speed also plays an important role in the dispersion parameter computations. At lower wind speeds, both initial vertical dispersion and vehicle-induced thermal effects lead to higher estimates of the vertical dispersion parameter and, hence, lower concentration estimates. In addition, wind speed will affect travel time to the measurement location, which can have an influence on (for example) the amount of coagulation for ultrafine particles. Wind speed can therefore have somewhat complex relationships with the spatial extent, due to sometimes competing effects on initial dilution, vertical dispersion, and for ultrafine particles, coagulation. Wind direction will also be influential, as whether the wind is parallel or perpendicular to the road (in upwind or downwind directions) will influence concentration patterns substantially.

In addition, according to the Gaussian equation above, a higher vertical dispersion coefficient (σ_z_) corresponds to lower downwind concentrations. Dispersion coefficients are functions of downwind distance and atmospheric stability. At the same downwind distance, unstable conditions correspond to higher dispersion coefficients than neutral conditions, followed by stable conditions. Stability classification in turn depends on measures of mechanical turbulence (such as surface roughness), measures of convective turbulence during daytime (such as mixing depth) and wind speed and wind direction fluctuations [24]. Therefore, if all other factors are the same, the spatial extent of influence for the same pollutant is smaller under unstable conditions.

To incorporate meteorological factors, we gathered information where possible on wind speed and direction. We coded each measurement as either being downwind from the road or upwind/parallel (with multiple estimates often available from an individual study as a function of wind direction). Wind speed was coded as above or below 2 m/s (the median value across studies). Few studies directly presented information on atmospheric stability or other meteorological factors, so we considered these factors only within our dispersion model application.

### Statistical analysis

To pool the evidence across selected studies, we considered two different dependent variables. The first is the reported spatial extent extracted from each of the studies. We evaluated predictors of the spatial extent in one-way and factorial ANOVAs. However, a number of studies reported no significant concentration gradient. Rather than omitting those studies from the analysis, we assigned each of these studies to have the maximum spatial extent reported across all studies of the same type (i.e., monitoring studies). Because of the potential that this approach could influence our findings, we also considered predictors of a dummy variable for above/below 500 meters.

### Case study

To corroborate the findings from the meta-analysis and to provide insight about the influence of multiple factors in a controlled setting, we conducted an illustrative case study. Assuming a flat terrain, we calculate the downwind incremental concentration (Ci) of a relatively inert pollutant A from a continuously emitting infinite line source, when the wind direction is perpendicular to the line, by Ci=2Q2πUσz
 MathType@MTEF@5@5@+=feaafiart1ev1aaatCvAUfKttLearuWrP9MDH5MBPbIqV92AaeXatLxBI9gBaebbnrfifHhDYfgasaacH8akY=wiFfYdH8Gipec8Eeeu0xXdbba9frFj0=OqFfea0dXdd9vqai=hGuQ8kuc9pgc9s8qqaq=dirpe0xb9q8qiLsFr0=vr0=vr0dc8meaabaqaciaacaGaaeqabaqabeGadaaakeaacqWGdbWqcqWGPbqAcqGH9aqpdaWcaaqaaiabikdaYiabdgfarbqaamaakaaabaGaeGOmaidcciGae8hWdahaleqaaOGaemyvauLae83Wdm3aaSbaaSqaaiabdQha6bqabaaaaaaa@39BE@ (as described above). The total concentration of this pollutant is the sum of the background (Cb) and incremental concentration (Ci):

Ctotal=Ci+Cb=2Q2πUσz+Cb
 MathType@MTEF@5@5@+=feaafiart1ev1aaatCvAUfKttLearuWrP9MDH5MBPbIqV92AaeXatLxBI9gBaebbnrfifHhDYfgasaacH8akY=wiFfYdH8Gipec8Eeeu0xXdbba9frFj0=OqFfea0dXdd9vqai=hGuQ8kuc9pgc9s8qqaq=dirpe0xb9q8qiLsFr0=vr0=vr0dc8meaabaqaciaacaGaaeqabaqabeGadaaakeaacqWGdbWqcqWG0baDcqWGVbWBcqWG0baDcqWGHbqycqWGSbaBcqGH9aqpcqWGdbWqcqWGPbqAcqGHRaWkcqWGdbWqcqWGIbGycqGH9aqpdaWcaaqaaiabikdaYiabdgfarbqaamaakaaabaGaeGOmaidcciGae8hWdahaleqaaOGaemyvauLae83Wdm3aaSbaaSqaaiabdQha6bqabaaaaOGaey4kaSIaem4qamKaemOyaigaaa@494E@

For the base case, we assume a source strength per unit distance of 5 μg/(m·s), wind speed of 4 m/s, neutral stability (Pasquill stability class D), and background concentration of zero. The vertical dispersion coefficient (σ_z_) can be calculated as σz2=σz,M2+σz,B2
 MathType@MTEF@5@5@+=feaafiart1ev1aaatCvAUfKttLearuWrP9MDH5MBPbIqV92AaeXatLxBI9gBaebbnrfifHhDYfgasaacH8akY=wiFfYdH8Gipec8Eeeu0xXdbba9frFj0=OqFfea0dXdd9vqai=hGuQ8kuc9pgc9s8qqaq=dirpe0xb9q8qiLsFr0=vr0=vr0dc8meaabaqaciaacaGaaeqabaqabeGadaaakeaaiiGacqWFdpWCdaqhaaWcbaGaemOEaOhabaGaeGOmaidaaOGaeyypa0Jae83Wdm3aa0baaSqaaiabdQha6jabcYcaSiabd2eanbqaaiabikdaYaaakiabgUcaRiab=n8aZnaaDaaaleaacqWG6bGEcqGGSaalcqWGcbGqaeaacqaIYaGmaaaaaa@3FB2@, combining the CALINE4 mixing zone calculation [28] (σ_z, M_) with a formula reflecting dispersion outside of the mixing zone [24] (σ_z, B_). The mixing zone is defined as the region over the traffic lanes plus three meters on either side. When wind is perpendicular to the line source, CALINE4 models the mixing zone vertical dispersion coefficient as σ_z, M_= 1.5+ 0.05 × W/U, in which W is the width of the mixing zone and U is the wind speed. Assuming a mixing zone width of 24 m and our base case wind speed, σ_z, M_= 1.8 m. Under stability class D, σ_*z, B *_= 0.06*x*(1+0.0015*x*)^-0.5^, in which × is the downwind distance from the edge of the mixing zone in meters.

With the above information, we calculate the downwind concentration at different distances from the source for different values of parameters deemed important in our meta-analysis (for inert pollutants only).

## Results

### Meta-analysis

In our literature review, we found 33 studies that met our selection criteria —18 monitoring studies [11,12,15,20,21,23,29-40], 4 air dispersion modeling studies[13,14,22,41], 1 land use regression study [9], 3 biomonitoring studies [42-44], and 7 epidemiology studies [1,5-8,45,46]. Three of these studies are regulatory reports [13,14,22], and the rest are from the peer-reviewed literature. All monitoring studies targeted automobile related air pollution from major highways or city roads, while air dispersion modeling studies investigated a wider range of ground level pollution sources including construction equipment, ports, locomotives and roadways. In addition, three of the four air dispersion modeling studies were regulatory reports, which also used a health risk framework in evaluating spatial extent. Additional files 1, 2, 3, 4, 5, 6  provide in detail the information extracted from the studies for particulate matter (PM) mass concentration, (ultrafine) particle number count, black smoke/black carbon/elemental carbon, CO and benzene, NO_x_, and all other pollutants/endpoints, respectively.

In total, these 33 studies yielded 67 estimates of spatial extent potentially suitable for quantitative meta-analysis. Estimates of spatial extent based on an "elevated cancer risk" definition were highly variable, based on the risk threshold used, and included some extreme values (8.5 km and 32 km, where the maximum for all other studies was 1000 m). As these values would significantly influence any meta-analyses, we excluded this category from future statistical analysis, while noting the substantive influence of this definition on spatial extent values. Of the remaining 63 estimates, three were based on dispersion modeling and eight on epidemiological studies, with the other 52 derived from monitoring studies. There was a moderately significant difference in spatial extent estimates by study type (p = 0.05), with a median spatial extent of 300 m in monitoring studies, versus 125 m in epidemiological studies and 400 m in dispersion modeling studies.

**Figure 1:**
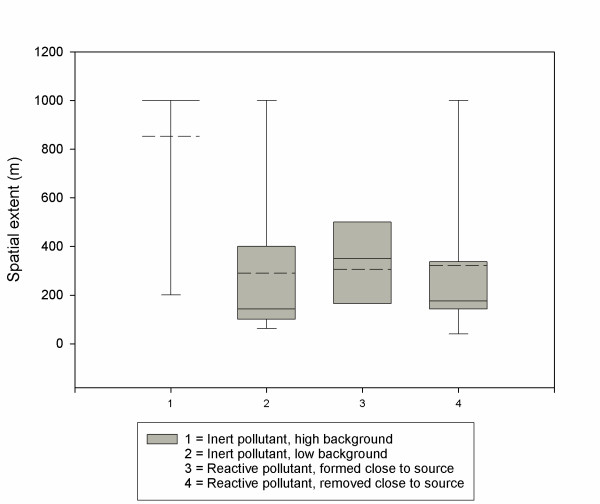
**Spatial extent estimates from studies in meta-analysis, stratified by pollutant type**. The dashed line represents the mean, the solid line the median, the box the 25^th^–75^th ^percentile range, and the whiskers the 5^th^–95^th ^percentage range.

There was a significant difference in spatial extent estimates by pollutant type (p < 0.0001, Figure 1), with a median value of 1000 m (the maximum value in the dataset) for inert pollutants with high background, versus 140 m for inert pollutants with low background, 350 m for reactive pollutants formed in the atmosphere, and 175 m for reactive pollutants removed from the atmosphere. The lack of a significant spatial gradient for inert pollutants with high background is driven in large part by studies of PM mass [11,15,31,32,34,36], in which the high background implies that spatial extent criteria based on percentage differences are never met (see additional file 1 – Particulate matter (PM) mass concentration related studies). The only studies in this category that did demonstrate significant spatial gradients were either land use regressions or monitoring studies using less traditional definitions of spatial extent (i.e., distance at which consecutive measurements were no longer significantly different from one another, distance after which representative sites for the average concentration are found). Of note, studies of PM mass that excluded background concentrations, e.g. monitoring results with background values subtracted [21] or air dispersion modeling studies focusing only on the source of interest [13,14,41], find significant concentration gradients.

In univariate ANOVA, the spatial extent definition was also a statistically significant predictor (p = 0.04), with significantly higher values among studies considering absolute differences from background levels (median of 500 m) rather than percentage differences from baseline levels or other approaches (medians of 180 m and 200 m, respectively). In addition, although the sample size was limited (n = 27), the spatial extent was significantly lower when the receptor was downwind from the road (p = 0.03). Wind speed was not a statistically significant predictor (p = 0.18), but the median spatial extent was greater under high wind conditions (425 m) than under low wind conditions (150 m). Traffic volume, as a proxy for emission rate, was a highly insignificant predictor (p = 0.60).

For our multivariate models, given the limited non-monitoring studies and the extent of missing information in those studies (no meteorological information and no pollutant characterization in epidemiological studies), we restrict our analysis to the 52 monitoring-based estimates, and focus on two-variable models given statistical power issues. In factorial ANOVA, pollutant type remains significant in all models, while definition of spatial extent becomes insignificant (p = 0.46) when controlling for pollutant type. Upwind/downwind conditions remained statistically significant even after controlling for pollutant type (p = 0.01). Conclusions were generally similar in logistic regression analyses considering spatial extent above/below 500 m.

### Case study

The meta-analysis clearly demonstrates the importance of pollutant type and background conditions, as well as meteorological factors to a lesser extent, but the literature is not sufficiently robust to capture the interactions among covariates (e.g., wind speed and stability class) or the effect of subtle variations in spatial extent definitions. In addition, the number of dispersion modeling studies found that satisfy all criteria is limited, therefore, running our simple dispersion model under a variety of conditions, we estimate the spatial extent using multiple definitions, the results of which are summarized in Table 1.

**Table 1:** Calculated spatial extent from illustrative case study under differing assumptions and definitions

**Factor under study**	**Emission rate (μg/m·s)**	**Background concentration (μg/m**^3^**)**	**Meteorological conditions**		**Definition of spatial extent**		**Estimated spatial extent (m)**
			
			**Wind speed (m/s)**	**Stability class**	**Comparison method**	**Threshold**	
Spatial extent definition	5	0	4	D	Absolute	0.25 μg/m^3^	70
	5	0	4	D	Absolute	0.1 μg/m^3^	190
	5	0	4	D	Absolute	0.05 μg/m^3^	430
	5	0	4	D	Relative	50% of reference	60
	5	0	4	D	Relative	20% of reference	170
	5	0	4	D	Relative	10% of reference	380
Emission rate	2.5	0	4	D	Absolute	0.1 μg/m^3^	90
	10	0	4	D	Absolute	0.1 μg/m^3^	430
Background concentration	5	0.25	4	D	Relative	50% of reference	120
	5	1	4	D	Relative	50% of reference	N/A
Meteorological factor	5	0	3	F	Absolute	0.25 μg/m^3^	350
	5	0	2	B	Absolute	0.25 μg/m^3^	70
	5	0	6	D	Absolute	0.25 μg/m^3^	40
Health risk threshold	5	0	4	D	Absolute	20 per million cancer risk	300
	5	0	4	D	Absolute	1 per million cancer risk	> 500 (beyond domain)

The spatial extent increases with the decrease in the threshold value for an absolute comparison. For example, if we define the concentration threshold value as 0.25, 0.1, or 0.05 μg/m^3^, the corresponding spatial extent increases from 70 to 190 to 430 m. Similarly, using a relative comparison to define the spatial extent, when we define the percentage threshold as 50, 20 and 10 percent of the reference concentration, the spatial extent increases from 60 to 170 to 380 m.

In addition, the emission rate can influence the spatial extent for absolute comparisons, with the spatial extent increasing from 90 m to 430 m when the emission rate increases from 2.5 to 10 μg/(m·s). Relative spatial extent definitions are unaffected by emission rates, at least with zero background concentrations. As the background concentration increases, the spatial extent based on a relative comparison increases correspondingly (Table 1). In an extreme case, when the background concentration is 1 μg/m^3^, the concentration never drops below 50% of the reference. Changing meteorology also clearly influences the spatial extent, with more unstable conditions (e.g., class B, D and F are moderately unstable, neutral and moderately stable respectively) resulting in lower spatial extents, although with an important modifying effect of wind speed (Table 1).

Finally, basing the spatial extent on cancer risk thresholds rather than concentrations significantly influences the spatial extent (Table 1). If we assume the pollutant under study is diesel PM, according to California EPA [47], the cancer risk potency factor is 300 per million per μg/m^3 ^over 70 years lifetime. The lifetime cancer risk would range from 166 to 13 per million from the edge of the mixing zone to 500 m downwind under the base case. The spatial extent corresponding to a threshold of 20 per million in cancer risk is about 300 m from the source, and the spatial extent for a threshold of 1 per million would be well beyond our modeling region. Of note, this definition corresponds directly with absolute concentration definitions, although with lower concentrations allowed (i.e., a 1 per million risk threshold corresponds with a 0.003 μg/m^3 ^concentration threshold).

## Discussion and conclusion

In this meta-analysis and case study, we have demonstrated that the substantial range in spatial extent estimates in the literature can be largely explained by three key factors – the definition of spatial extent used, the characteristics of the pollutant of interest, and the local meteorology. Extremely large spatial extent values are seen when using a risk threshold definition, especially when the threshold is relatively low (i.e., a 1 in a million cancer risk). Setting that approach aside, the remainder of the literature is reasonably consistent. Non-reactive pollutants with high background concentrations (i.e., PM mass) demonstrate little discernable spatial gradient, and there is then a general hierarchy based on whether a pollutant is formed or removed from the atmosphere in the short term (Figure 1). The smallest spatial extent estimates occur for pollutants like ultrafine particles and NO and when wind speeds are lower. These meta-analysis findings agree with our dispersion modeling results, which further demonstrated lower spatial extent estimates for spatial extent definitions with high thresholds, for low emission rates, and for more unstable meteorological conditions.

While it is apparent based on first principles that many factors can affect the spatial extent of influence for mobile sources, the relative contributions of these factors and importance of definitions as well as pollutant type and site/meteorological characteristics have not been previously considered. The spatial extent definition was not statistically significant in multivariate models, but it was clearly influential in our case study, especially when the risk-based estimates are considered or subtleties in the definitions are evaluated. There is no single commonly accepted definition regarding the comparison method, threshold value, or inclusion of background concentration; the appropriate choices will be driven in part by the question under study. For example, when a regulatory threshold is involved, an absolute comparison is normally used, in which concentrations/health risks at different distances are compared with the regulatory related threshold, e.g., 1 in a million health risk. Other studies, e.g., those focusing on the behavior of specific pollutants, utilize relative comparisons more often, as it helps to remove the influence of factors such as meteorology and emissions when combining monitoring results on different days. In addition to comparison method, a wide range of threshold values are used (ranging from 10 to 50 percent of the maximum concentration in relative comparisons in the studies reviewed), and studies address background concentrations in different ways. Many of these decisions were somewhat arbitrary within published studies, yet these factors (especially treatment of background concentrations) explain a substantial portion of the variability in findings.

In spite of the above intricacies, the literature allows us to develop some first-order rules of thumb for policy makers and other stakeholders. Omitting the health risk threshold perspective or circumstances with high background concentrations and no significant gradients, the spatial extent of impact for mobile sources reviewed in this study is generally on the order of 100–400 m for elemental carbon or particulate matter mass concentration (excluding background concentration), 200–500 m for NO_2_, and 100–300 m for ultrafine particle count. From a policy perspective, this might indicate that a 500 meter buffer around a roadway would be appropriately protective under most circumstances. However, policy makers may be concerned about risk thresholds, which could imply quite large spatial extents of impact. While these distances could be implausibly large for offsets/buffers, this alternative framing emphasizes that there are circumstances in which exposure increments that are difficult to detect and well below maximum impacts may still be relevant for public health, and studies with an individual health risk framing should not restrict their focus to a 500 meter radius.

In addition, it should be noted that all studies within our meta-analysis defined spatial extent based on either concentration or individual health risk. However, within benefit-cost analyses and related applications, total population exposure (the product of population and incremental concentration change) would be the endpoint of interest, and an alternative definition of spatial extent would consider the distance from the source by which a substantial fraction of the total population exposure was achieved. This definition of spatial extent has been used in previous studies of power plant health risks [48-51] and has been developed for mobile sources as well [52]. Under this definition, the distance of concern will depend on population patterns, and could be significantly greater than under other definitions (i.e., if few people lived close to the roadway but population density was high at longer range).

There are some limitations in the conclusions that can be drawn from our meta-analysis and case study. As mentioned before, one limitation is that many of the papers reviewed do not directly focus on spatial extent. In gathering necessary information we either contacted the authors of these studies for clarification/additional information or used our judgments to determine proxies of spatial extent in cases where direct information was not available. More broadly, many important factors were not reported in these publications; fewer than half of estimates included information on wind speed or direction, limiting our ability to conduct robust multivariate analyses. The studies also varied substantially in the pollutants measured, methods used, and site characteristics, and our simple categorical variables may have omitted some important nuances. The number of studies was insufficient to look in more detail at, for example, differences across different particulate matter size fractions and definitions. We were also constrained to the configurations previously evaluated in the literature, along with a simple configuration in our case study. Other circumstances (i.e., an elevated highway or street canyon) could yield somewhat different conclusions about the spatial extent, so our findings are not generalizable to all circumstances.

In spite of these limitations, we can draw some useful conclusions both for future research on the spatial extent of mobile source air pollution and for policymakers and stakeholders interested in utilizing this concept. First, to allow for meaningful comparisons across studies, it is important to state the definition of spatial extent explicitly, including in particular the comparison method, threshold values, and whether background concentration is included. While many monitoring studies are not conducted explicitly for this purpose, the authors should at a minimum take care to define terms before making statements about the distance over which impacts were observed. Second, the observation that the spatial extent is generally within a few hundred meters for highway or city roads demonstrates the need for high resolution modeling near the source for studies focused on exposure patterns or individual risk thresholds. The question regarding the appropriate resolution and scope for benefit-cost analysis was not addressed in our study, but our findings confirm the expectation that important dynamics within hundreds of meters of the roadway may be influential for population exposure as well, at least in dense urban areas. Finally, our findings emphasize that policymakers should be able to develop reasonable estimates of the "zone of influence" of mobile sources, provided that they can clarify the pollutant of concern, the general site characteristics, and the underlying definition of spatial extent that they wish to utilize.

## Competing interests

The author(s) declare that they have no competing interests.

## Authors' contributions

YZ carried out the literature search and review, coded the relevant information for the meta-analysis, conducted initial statistical analysis and drafted the manuscript. JIL conceived of the study, refined the meta-analysis and revised the manuscript. All authors read and approved the final manuscript.

## Pre-publication history

The pre-publication history for this paper can be accessed here:



## Supplementary Material

Additional file 1 Particulate matter (PM) mass concentration related studies Click here for file

Additional file 2 Particle number count/ultrafine particle count related studies Click here for file

Additional file 3 Black smoke/Black carbon/Elemental carbon related studies Click here for file

Additional file 4 CO and benzene related studies Click here for file

Additional file 5 NO_2_, NO, NO_x _related studies Click here for file

Additional file 6 Non pollutant specific studies Click here for file
