# Supplementary material for: Factors influencing the spatial extent of mobile source air pollution impacts: a meta-analysis
Source: BMC Public Health. 2007 May 22;7:89. doi: 10.1186/1471-2458-7-89 (PMC1890281; doi:10.1186/1471-2458-7-89)
Supplement: Additional file 1 — Particulate matter (PM) mass concentration related studies [file 1471-2458-7-89-S1.doc]

Table 1 Particulate matter (PM) mass concentration related studies

| **Study** | **Location /season** | **Study /source type** | **Background** | **Emission rate/ traffic volume** | **Pollutant** | **Meteorology (wind speed/ direction/ stability)** | **Definition of spatial extent** | **Result** |
| --- | --- | --- | --- | --- | --- | --- | --- | --- |
| **[14]** | Roseville Rail Yard, CA, US/ 1 year | Air dispersion model/Diesel fueled locomotives | For Sacramento region, the estimated background risk level from diesel PM is 360 in a million and 520 in a million for all toxic air pollutants | 30,000 locomotives visit the Yard annually; 25 tons per year diesel PM emissions in 2000 | Diesel PM | Meteorology data from ARB air quality monitoring station at Roseville within one mile from the site. Also test data from another station 10 miles southwest of the site in the sensitivity analysis | Elevated cancer risk | Area with cancer risk greater than 500 in a million: 10 to 40 acres (114 to 227m radius); 10 to 100 in a million: 46,000 to 56,000 acres (7,698 to 8,493m radius) |
| **[14]** | Roseville, CA, US/1 year | Air dispersion model/highway |  | 10,000 heavy-duty diesel trucks/day (417 vehicles/h) | Diesel PM | Meteorology data from ARB air quality monitoring station at Roseville | Elevated cancer risk | 10 in a million risk: 1 mile (1,609m); 100 in a million risk: 300 feet (91m) |
| **[13]** | Ports of Los Angeles and Long Beach, CA, US/ 1 year | Air dispersion model/port | Not included | 965 tons/year for POLA; 795 tons/year for POLB in 2002 | Diesel PM | Wind speed and direction, temperature, stability data etc from meteorology station one mile away from the ports | Elevated cancer risk | 20mile (32km) |
| **[9]** | The Netherlands, Munich, Germany and Sweden/one year | Regression | Background sites have no more than 3000 vehicles/day pass through a circle with 50m radius around the site | Traffic sites have greater than 3000 vehicles/day within 50m radius (125 vehicles/h) | PM_2.5_ |  | Traffic-related variables involving spatial extent chosen in the final regression model to predict concentrations measured at monitoring sites | Variable traffic density within 250m buffer was chosen in the regression |
| **[41]** | Portland, OR, US | Air dispersion model and regression/all road types | Intercept term in the regression model | Emissions estimated based on travel demand from EMME2 and emission factors from MOBILE6.2 | Diesel PM | 16 possible wind directions and 7 possible wind speeds incorporated in emission contribution estimates | Buffer radius for which regression model predicting CALPUFF concentrations using emission contributions performed best | 200-400m |
| **[29]** | Seattle, WA, US/ 4 seasons | monitor and regression/arterial roads | Overall average concentration of all sites on a particular day | average daily traffic count of 3000 cars or greater (125 vehicles/h) | PM_2.5_ |  | Distance after which representative sites for the overall average concentration are found | 100-300m |
| **[15]** | Australia | Monitor/major road | ambient monitor | 2,130-3,400 vehicles/h | PM_2.5_ | Wind from road to receptors | 50% of maximum concentration | N/A, 75% of maximum at 375m |
| **[15]** | Australia | Monitor/major road | ambient monitor | 2,130-3,400 vehicles/h | PM_2.5_ | Wind parallel to road | 50% of maximum concentration | N/A, 65% of maximum at 375m |
| **[30]** | UK | Monitor/major artery road | Houses more than 50m from major road | 1,200-2,500 vehicles/h | PM_10_ |  | statistically significant difference between 'proximity' and 'background' homes | N/A |
| **[30]** | UK | Monitor/major artery road | Houses more than 50m from major road | 1,200-2,500 vehicles/h | PM_2.5_ |  | statistically significant difference between 'proximity' and 'background' homes | N/A |
| **[31]** | Boston, MA, US/ July and August | monitor and regression/city roads | 25 m in an upwind direction | 96-1,150 vehicles/h | PM_2.5_ | wind speed 0.4 to 8.2 m/s | Concentration gradient over distance of 80m; significance of traffic density score in predicting concentration across different radii | Slight gradient; traffic density score not significant in predicting concentration across radii from 50 to 300m |
| **[32]** | Zurich, Switzerland/ November to January and June to August | Monitor/City road with moderate traffic | Measurement at 20 m above ground | 8,800 vehicles/day (367 vehicles/h) | PM_10_ |  | Percentage of maximum measured at the road | N/A, 10 to 15% decrease between road and 15m, level off after 15m |
| **[33]** | Cincinnati, US | Monitor/  highway | 1600 m from the highway | 135,000 to 150,000 vehicles/day (5625 to 6250 vehicles/h) | PM_2.5_ | Wind from highway towards sampling locations 60% of the time, average wind speed of 1.8 m/s | Difference from the background | N/A, only 20% decrease from 80 to 1600 m |
| **[34]** | Province of South Holland, the Netherlands/ May to July | Monitor/major motorway | Most far away monitors at 260 to 305m* | 80,000 to 152,000 vehicles/day (3333 to 6333 vehicles/h) | PM_10_ | High exposure if wind was within 60 degree from perpendicular to the road in the direction of the city district under study at least 33% of the time | Concentration gradient along distance | No gradient |
| **[34]** | Province of South Holland, the Netherlands / May to July | Monitor/major motorway | Most far away monitors at 260 to 305m* | 80,000 to 152,000 vehicles/day (3333 to 6333 vehicles/h) | PM_2.5_ | High exposure if wind was within 60 degree from perpendicular to the road in the direction of the city district under study at least 33% of the time | Concentration gradient along distance | No gradient |
| **[22]** | Consumes Power Plant in CA, US | Air dispersion model/Construction combustion and fugitive dust emissions |  | Max daily emissions is 36.7 lb/day; annual emissions is 1.9 ton/year | PM_10_ | Used the Elk Grove, Stockton Blvd, Del Paso Manor, and T Street monitoring stations for 1997 to 2000 to establish the ambient background levels | Concentration gradient; elevated cancer risk for the diesel exhaust portion of the PM_10_ | 200 m to reach nearly 80% concentration reduction; 300m in which cancer risk may exceed 1 in a million |
| **[35]** | Kuopio, Central Finland/ August to September | monitor/major road | Weighted (by inverse distance) average of concentrations measured at EMEP stations | 17,000 to 22,000 vehicles/day (718 to 917 vehicles/h) | PM_2.5_ | Wind speed at 10 m averaged 2.6m/s, atmospheric stratification varied from very unstable to stable; mixing height from 100 to 3000 m | Difference between concentrations measured at two consecutive distances no longer statistically significant | 52 to 87m; 30% decrease over 87m. |
| **[21]** | The Netherlands | Monitor/Busy route | Urban background, which is removed from measurement | more than 10,000 vehicles/day (417 vehicles/h) | PM_>1_ |  | 90% decrease from maximum concentration | 75m |
| **[21]** | The Netherlands | Monitor/Busy route | Urban background, which is removed from measurement | more than 10,000 vehicles/day (417 vehicles/h) | PM_1_ |  | 90% decrease from maximum concentration | 120m |
| **[36]** | Macao / December | Monitor/Major roads | N/A | 1,633/h | PM_1_ | Northerly wind for from road to measuring sites for 2 days and a half; southeasterly wind for half a day. Wind speed from 1.4 to 9.2 m/s | Percentage of maximum concentration at 2m from the road | N/A, 10% decrease over 228 m |
| **[36]** | Macao/ December | Monitor/Major roads | N/A | 1,633/h | PM_10_ | Northerly wind for from road to measuring sites for 2 days and a half; southeasterly wind for half a day. Wind speed from 1.4 to 9.2 m/s | Percentage of maximum concentration at 2m from the road | N/A, 7% decrease over 228 m |
| **[36]** | Macao/ December | Monitor/Major roads | N/A | 1,633/h | PM_2.5_ | Northerly wind for from road to measuring sites for 2 days and a half; southeasterly wind for half a day. Wind speed from 1.4 to 9.2 m/s | Percentage of maximum concentration at 2m from the road | N/A, 9% decrease over 228 m |
| **[11]** | Southern CA, US/ May to July | Monitor/freeway | Upwind monitor | 13,900 vehicles/h | PM mass | Wind from road to receptors most of the sampling time with a speed of 1-2 m/s | 60% decrease from maximum concentration | N/A, only a few percent decrease over 300m |

Note: under “Emission rate/traffic volume” column, traffic count in parenthesis in vehicles/hour is converted from vehicles/day assuming 24 hours/day.
