# Supplementary material for: Factors influencing the spatial extent of mobile source air pollution impacts: a meta-analysis
Source: BMC Public Health. 2007 May 22;7:89. doi: 10.1186/1471-2458-7-89 (PMC1890281; doi:10.1186/1471-2458-7-89)
Supplement: Additional file 2 — Particle number count/ultrafine particle count related studies [file 1471-2458-7-89-S2.doc]

Table 2 Particle number count/ultrafine particle count related studies

| **Study** | **Location /season** | **Study /source type** | **Background** | **Emission rate/traffic volume** | **Pollutant** | **Meteorology (wind speed/ direction/ stability)** | **Definition of spatial extent** | **Result** |
| --- | --- | --- | --- | --- | --- | --- | --- | --- |
| **[15]** | Australia | Monitor/major road | ambient monitor | 2,130-3,400 vehicles/h | ultrafine particle count | Wind from road to receptors | 50% of maximum concentration | 100-150 m |
| **[15]** | Australia | Monitor/major road | ambient monitor | 2,130-3,400 vehicles/h | ultrafine particle count | Receptors upwind from road | 50% of maximum concentration | No effect |
| **[15]** | Australia | Monitor/major road | ambient monitor | 2,130-3,400 vehicles/h | ultrafine particle count | Wind parallel to road | 50% of maximum concentration | 50-100 m |
| **[31]** | Boston, MA, US/ July and August | monitor and regression/city roads | 25 m in an upwind direction | 96-1,150 vehicles/h | ultrafine particle number | wind speed 0.4 to 8.2 m/s | Concentration gradient over distance of 80m; significance of traffic density score in predicting concentration across different radii | 100 to 200m for which traffic density score significant in predicting concentration; significant distance gradient to 50m |
| **[37]** | Australia | Monitor/freeway | ambient monitor |  | particle number count | 0.1 m/s from road to receptors in the morning changing to 2m/s from receptors to road from mid morning through afternoon | statistically significant decrease in particle number count with distance | <60m |
| **[20]** | Helsinki, Finland | Monitor/highway | 600m northwest of the highway | 3,065 vehicles/h during morning rush hour | particle number count | prevailing wind direction northwest; wind speed mostly about 2m/s and always below 4m/s | 10% of the maximum values measured at 50m from the source | 120-140m |
| **[33]** | Cincinnati, US | Monitor/highway | 1600 m from the highway | 135,000 to 150,000 vehicles/day (5625 to 6250 vehicles/h) | ultrafine particle count | Wind from road to receptors, average wind speed 1.3 m/s | 50% of the maximum values measured at 9m from the source | 150 m |
| **[33]** | Cincinnati, US | Monitor/highway | 1600 m from the highway | 135,000 to 150,000 vehicles/day (5625 to 6250 vehicles/h) | ultrafine particle count | Wind parallel to road, average wind speed 0.6 m/s | 50% of the maximum values measured at 9m from the source | NA, still at 80% at 400m |
| **[21]** | The Netherlands | Monitor/Busy route | Urban background, which is removed from measurement | more than 10,000 vehicles/day (417 vehicles/h) | particle number count |  | 90% decrease from maximum concentration | 150m |
| **[12]** | Southern CA, US/ August to October | Monitor/freeway | Upwind monitor | 12,180 vehicles/h | ultrafine particle count | Wind from road to receptors 80% of time with wind speed <3 m/s, average wind speed 1.5m/s | 60 to 80% decrease from maximum concentration; statistically within variation of upwind concentration | 100m; 150 to 300m |
| **[11]** | Southern CA, US/ May to July | Monitor/freeway | Upwind monitor | 13,900 vehicles/h | ultrafine particle count | Wind from road to receptors most of the sampling time with a speed of 1-2 m/s | 60% decrease from maximum concentration; indistinguishable from upwind concentration | 100m; 300m |
