# Supplementary material for: Factors influencing the spatial extent of mobile source air pollution impacts: a meta-analysis
Source: BMC Public Health. 2007 May 22;7:89. doi: 10.1186/1471-2458-7-89 (PMC1890281; doi:10.1186/1471-2458-7-89)
Supplement: Additional file 3 — Black smoke/Black carbon/Elemental carbon related studies [file 1471-2458-7-89-S3.doc]

Table 3 Black smoke/Black carbon/Elemental carbon related studies

| **Study** | **Location /season** | **Study /source type** | **Background** | **Emission rate/traffic volume** | **Pollutant** | **Meteorology (wind speed/direction/stability)** | **Definition of spatial extent** | **Result** |
| --- | --- | --- | --- | --- | --- | --- | --- | --- |
| **[9]** | The Netherlands, Munich, Germany and Sweden/one year | Regression | Background sites have no more than 3000 vehicles/day pass through a circle with 50m radius around the site | Traffic sites have greater than 3000 vehicles/day within 50m radius (125 vehicles/h) | filter absorbance |  | Traffic-related variables involving spatial extent chosen in the final regression model to predict concentrations measured at monitoring sites | Variable traffic density within 250m buffer was chosen in the regression |
| **[30]** | UK | Monitor/ major artery road | Houses more than 50m from major road | 1,200-2,500 vehicles/h | absorbance of PM_10_ |  | statistically significant difference between 'proximity' and 'background' homes | N/A |
| **[30]** | UK | Monitor/ major artery road | Houses more than 50m from major road | 1,200-2,500 vehicles/h | absorbance of PM_2.5_ |  | statistically significant difference between 'proximity' and 'background' homes | 50m (borderline significant at p= 0.05 level) |
| **[34]** | Province of South Holland, the Netherlands/ May to July | Monitor/ major motorway | Most far away monitors at 260 to 305 m | 80,000 to 152,000 vehicles/day (3333 to 6333 vehicles/h) | black smoke | High exposure if wind was within 60 degree from perpendicular to the road in the direction of the city district under study at least 33% of the time | Concentration gradient along distance | 110 to 165m |
| **[12]** | Southern CA, US/ August to October | Monitor/ freeway | Upwind monitor | 12,180 vehicles/h | BC | Wind from road to receptors 80% of time with wind speed <3 m/s, average wind speed 1.5m/s | 60 to 80% decrease from maximum concentration | 100m |
| **[11]** | Southern CA, US/ May to July | Monitor/ freeway | Upwind monitor | 13,900 vehicles/h | BC | Wind from road to receptors most of the sampling time with a speed of 1-2 m/s | 60% decrease from maximum concentration | 100m |
