# Supplementary material for: Factors influencing the spatial extent of mobile source air pollution impacts: a meta-analysis
Source: BMC Public Health. 2007 May 22;7:89. doi: 10.1186/1471-2458-7-89 (PMC1890281; doi:10.1186/1471-2458-7-89)
Supplement: Additional file 5 — NO2, NO, NOx related studies [file 1471-2458-7-89-S5.doc]

Table 5 NO_2_, NO, NO_x_ related studies

| **Study** | **Location /season** | **Study /source type** | **Background** | **Emission rate/traffic volume** | **Pollutant** | **Meteorology (wind speed/direction/stability)** | **Definition of spatial extent** | **Result** |
| --- | --- | --- | --- | --- | --- | --- | --- | --- |
| **[39]** | Canada/ September | Monitor/highway | Measurement upwind (west of) the highway | 185,000 vehicles/day (7,708 vehicles/h) | NO_2_ | Wind from west | Major NO_2_ decrease | 200m |
| **[32]** | Zurich, Switzerland / November to January and June to August | Monitor/City road with moderate traffic | Measurement at 20 m above ground | 8,800 vehicles/day (367 vehicles/h) | NO_2_ |  | Percentage of maximum measured at the road | Greater than or equal to 80m in the summer; less than 10% decrease over 80m in the winter |
| **[40]** | South-west Sweden | Monitor/ highway | Measurement 300m upwind (west of) the highway | 18,900 to 32,500 vehicles/day (788 to 1354 vehicles/h) | NO_2_ | Wind from west | Contribution from highway becomes negligible | 500m |
| **[23]** | Southern CA, US/ July to September | Monitor/ highway | 30m upwind from the highway | 200,000 vehicles/day (8,333 vehicles/h) | NO | Wind speed 1.3-2.6m/s and directions within +- 45°arc sector of perpendicular to freeway | Less than 0.01 ppm influence on ambient measurement | 150-350m |
| **[23]** | Southern CA, US/ July to September | Monitor/ highway | 30m upwind from the highway | 200,000 vehicles/day (8,333 vehicles/h) | NO_2_ | Wind speed 1.3-2.6m/s and directions within +- 45°arc sector of perpendicular to freeway | Less than 0.01 ppm influence on ambient measurement | 500m |
| **[34]** | Province of South Holland, the Netherlands /May to July | Monitor/ major motorway | Most far away monitors at 260 to 305m* | 80,000 to 152,000 vehicles/day (3,333 to 6,333 vehicles/h) | NO_2_ | High exposure if wind was within 60 degree from perpendicular to the road in the direction of the city district under study at least 33% of the time | Concentration gradient along distance | 110 to 165m |
| **[38]** | Northern California, US/Spring and Fall | Monitor/ highway | Schools upwind or more than 1000 m downwind from freeway | 90,000 to 210,000 vehicles/day (3,750 to 8,750 vehicles/h) | NO_2_, NO_x_ | Wind from west or southwest during the day, mean wind speed from 3 to 6 m/s | Concentration gradient along distance | 350m, mentioned the near traffic effects more pronounced for NO_x_ |
| **[42]** | Scotland, UK/ 1 year | Biomonitor/ all road types | Sites farther away from the road | 1,000 to 50,000 vehicles/day (42 to 2,083 vehicles/h) | NO_x_ | Prevailing south-westerly wind | Gradient of NO_x_ concentration and Ellenberg fertility indices of the vegetation communities | >=11m |
