# Supplementary material for: Factors influencing the spatial extent of mobile source air pollution impacts: a meta-analysis
Source: BMC Public Health. 2007 May 22;7:89. doi: 10.1186/1471-2458-7-89 (PMC1890281; doi:10.1186/1471-2458-7-89)
Supplement: Additional file 6 — Non pollutant specific studies [file 1471-2458-7-89-S6.doc]

Table 6 Non pollutant specific studies

| **Study** | **Location /season** | **Study /source type** | **Background** | **Emission rate /traffic volume** | **Pollutant /endpoints under study** | **Meteorology (wind speed /direction /stability)** | **Definition of spatial extent** | **Result** |
| --- | --- | --- | --- | --- | --- | --- | --- | --- |
| **[43]** | Southern England | Biomonitor /highway | Where vegetation became more uniform; where little further change in plant species composition with increasing distance observed | 800 to 34,661 vehicles/12-hr (67 to 2,888 vehicles/h) | Species composition |  | Trends in the species composition | <=200m depending on traffic density |
| **[45]** | The Netherlands/ June | Epidemiology /major motorway |  | 80,000 to 150,000 vehicles/day (3,333 to 6,250 vehicles/h) | Lung function in children |  | Effect modification by distance on the association between lung function and truck traffic density | 300m |
| **[8]** | MA, US | Epidemiology /Highway and major road | More than 400 m away from a major road | 9,351 vehicles/day (390 vehicles/h) | persistent wheeze;chronic phlegm |  | Statistically significant adjusted odds ratio | 50 m |
| **[7]** | The Netherlands | Epidemiology /Highway and major urban road | Measured regional background concentration and estimated urban background concentration |  | Cardiopulmonary mortality; NO_2_ and black smoke used as indicators of traffic related air pollution |  | Statistically significant association between cardiopulmonary mortality and living near a major road | 100m within highway; 50m within major road |
| **[5]** | New York State, US | Epidemiology /state route |  | Vehicle miles traveled within 200m 4,595 on average | asthma hospitalization among children 0 to 14 year |  | Statistically significant adjusted odds ratio between case and control | 200m and with heavy traffic density |
| **[44]** | Rome, Italy / April to August and October | Biomonitor/major traffic axes, roundabout square | Green areas, e.g., adjacent park | High traffic roads have intensive traffic with usual traffic jams | magnetic susceptibility |  | 80% decrease in magnetic susceptibility | 25m |
| **[23]** | Southern CA, US / July to September | Monitor /highway | 30m upwind from the highway | 200,000 vehicles/day (8,333 vehicles/h) | O_3_ | wind speed 1.3-2.6m/s and directions within +- 45°arc sector of perpendicular to freeway | Less than 0.01 ppm influence on ambient measurement | 500m |
| **[1]** | Province of South Holland, the Netherlands / May to July | Epidemiology /highway |  | 80,000 to 150,000 vehicles/day (3,333 to 6,250 vehicles/h) | Cough, wheeze, runny nose, and doctor diagnosed asthma among children 7 to 12 year | Downwind defined as a wind direction of 120 degree around a line perpendicular to the freeway | Statistically significant association between number of symptoms and distance | 100m |
| **[6]** | Nottingham, UK | Epidemiology /main road | 150 m away from a major road | 10,000 to 100,000 vehicles/day (417 to 4,167 vehicles/h) | risk of wheeze among children 4 to 16 year |  | Statistically significant trend in adjusted odds ratio per 30-m increment | 150m, primarily within 90 m |
| **[46]** | Jimma, Ethiopia | Epidemiology /main road | 150 m away from a major road | 655 vehicles/12-h (55 vehicles/h) | risk of wheeze in adults and children |  | Statistically significant trend in adjusted odds ratio per 30-m increment | <150m |
